# Ex Vivo ^13^C-Metabolic Flux Analysis of Porcine Circulating Immune Cells Reveals Cell Type-Specific Metabolic Patterns and Sex Differences in the Pentose Phosphate Pathway

**DOI:** 10.3390/biom14010098

**Published:** 2024-01-12

**Authors:** Melanie Hogg, Eva-Maria Wolfschmitt, Ulrich Wachter, Fabian Zink, Peter Radermacher, Josef Albert Vogt

**Affiliations:** Institute for Anesthesiological Pathophysiology and Process Engineering, Ulm University Medical Center, 89081 Ulm, Germany; eva-maria.wolfschmitt@uni-ulm.de (E.-M.W.); ulrich.wachter@uni-ulm.de (U.W.); fabian.zink@uni-ulm.de (F.Z.); peter.radermacher@uni-ulm.de (P.R.); josef.vogt@uni-ulm.de (J.A.V.)

**Keywords:** aldosterone, Bayesian modeling, gas chromatography-mass spectrometry, granulocytes, ^13^C-labeling pattern, mitochondrial respiration, peripheral blood mononuclear cells, reactive oxygen species, tricarboxylic acid cycle

## Abstract

In general, females present with stronger immune responses than males, but scarce data are available on sex-specific differences in immunometabolism. In this study, we characterized porcine peripheral blood mononuclear cell (PBMC) and granulocyte energy metabolism using a Bayesian ^13^C-metabolic flux analysis, which allowed precise determination of the glycolytic, pentose phosphate pathway (PPP), and tricarboxylic acid cycle (TCA) fluxes, together with an assessment of the superoxide anion radical (O_2_^•−^) production and mitochondrial O_2_ consumption. A principal component analysis allowed for identifying the cell type-specific patterns of metabolic plasticity. PBMCs displayed higher TCA cycle activity, especially glutamine-derived aspartate biosynthesis, which was directly related to mitochondrial respiratory activity and inversely related to O_2_^•−^ production. In contrast, the granulocytes mainly utilized glucose via glycolysis, which was coupled to oxidative PPP utilization and O_2_^•−^ production rates. The granulocytes of the males had higher oxidative PPP fluxes compared to the females, while the PBMCs of the females displayed higher non-oxidative PPP fluxes compared to the males associated with the T helper cell (CD3^+^CD4^+^) subpopulation of PBMCs. The observed sex-specific differences were not directly attributable to sex steroid plasma levels, but we detected an inverse correlation between testosterone and aldosterone plasma levels and showed that aldosterone levels were related with non-oxidative PPP fluxes of both cell types.

## 1. Introduction

The cell-mediated immune response may present with sex-specific differences, inasmuch as females produce more vigorous cellular and humoral immune reactions and, thus, tend to be more resistant against stress and bacterial infection [1,2,3]. Previous reports indicated that sex hormones, such as estrogen and testosterone, have immunomodulatory effects on peripheral immune cells, which may be mediated through sex hormone receptors both on peripheral blood mononuclear cells (PBMCs) and neutrophils [4,5,6,7,8]. This highlights the importance of taking sex as a crucial biological variable into consideration when assessing cell type-specific metabolic pathways that may impact immune cell function. Silaidos et al. reported higher mitochondrial respiration in PBMCs originating from healthy women compared to men [9], while neutrophils from men showed a higher O_2_ consumption rate (OCR) without any difference in glycolysis as determined by the extracellular acidification rate (ECAR). However, the interpretation of the OCR/ECAR ratio does not allow a quantitative relation to any metabolic flux [10] and does not provide information about glutamine-driven metabolism or the pentose phosphate pathway (PPP). Hence, detailed reports on sex-specific differences in the glycolytic and tricarboxylic acid (TCA) cycle pathways are limited despite the clear evidence that these metabolic pathways are essential for immune cell function and differentiation.

As cells of the innate immune system and the most frequent leukocytes in peripheral blood, granulocytes form the first line defense against pathogens. They are recruited to sites of injury or infection by inflammatory signals and produce a variety of effector functions when activated, including neutrophil extracellular traps (NETs) formation, phagocytosis, and oxidative burst. PBMCs, on the other hand, are a fraction of mononuclear immune cells that are involved in both humoral and cellular defense. They comprise monocytes and lymphocytes (T cells, B cells, and natural killer cells) and, therefore, represent cells of both innate and adaptive immunity. Due to their accessibility in circulation and involvement in health and disease, PBMCs have been suggested as potential biomarkers, e.g., in heart failure and septic shock [11,12,13,14,15]. In contrast to granulocytes, which require mainly glycolytic pathways for energy production and antimicrobial defense [16,17], PBMCs have a higher mitochondrial O_2_ consumption and primarily utilize glutamine via the TCA cycle for proliferation, differentiation, and the effector function [18]. In immune cells, the PPP has been shown to be a key regulator of cellular mechanisms like proliferation and survival [19,20,21]. It provides both ribose-5-phosphate (R5P) for nucleotide biosynthesis and NADPH as a substrate for the NADPH oxidase to promote reactive oxygen species (ROS) production. Recently, in vitro studies have demonstrated that stimulation with sex steroids prevented superoxide anion (O_2_^•−^) production in human neutrophils [5,22,23]; however, it is still unclear whether these effects are already present in resting neutrophils, i.e., at physiological steroid levels. Moreover, it is an open question whether reduced ROS production is associated with decreased activity of the oxidative PPP, the main source of NADPH in granulocytes.

While the metabolic concentration provides no information about pathway activity in a complex and dynamic reaction network, metabolic flux analysis (MFA) is a powerful approach that can yield absolute values for intracellular metabolic fluxes [24,25]. For ^13^C-MFA, cells are incubated with ^13^C-labeled substrates (e.g., [U-^13^C]glucose, [U-^13^C]glutamine). The following incorporation of labeled ^13^C atoms generates distinct labeling patterns of metabolites depending on which pathways are used [26]. For example, the metabolism of [1,2-^13^C]glucose through the combined oxidative and non-oxidative PPP results in single-labeled lactate, while utilization of the glycolysis pathway alone leads to intermediates with double labeling only [27]. Until now, gas chromatography-mass spectrometry (GC-MS) has been the preferred analytical technique to determine ^13^C-labeling patterns of intracellular metabolites and their fragments, especially TCA cycle intermediates [28,29], while liquid chromatography-mass spectrometry (LC-MS) has been the most commonly used technique to detect PPP metabolites, such as sugar phosphates [19,30,31,32]. However, determination of the PPP is challenging due to the reversibility of the non-oxidative PPP and its interconnection with several metabolic pathways, such as glycolysis/gluconeogenesis and the glycogen synthesis/degradation cycle. Recently, we proposed a novel GC-MS-based Bayesian ^13^C-MFA for precise determination of glucose metabolism that enables the simultaneous quantification of glycolytic, PPP, and TCA cycle fluxes ex vivo. The Bayesian approach additionally provides pairwise correlations of fluxes with their joint confidence regions, which provide valuable information regarding cellular mechanisms [33].

In this study, we applied our Bayesian ^13^C-MFA to unstimulated PBMCs and granulocytes from healthy pigs of either sex for characterization of their immunometabolism [33,34]. Furthermore, we included the total oxygen consumption and the O_2_^•−^ production to investigate the interrelations between metabolic pathways. Principal component analysis (PCA) is a method of data exploration that reduces the dimensionality of a dataset by identifying linear combinations between the analyzed parameters that retain the largest variance in the dataset. Thus, we identified which metabolic fluxes (i) show strong relations with other fluxes and (ii) explain the main variability in the metabolic profile. Furthermore, the visualization of the PCA allowed us to assess similarities and differences in the metabolic state among the samples and to identify the unknown factors, e.g., sex or cell population, that impact metabolic patterns.

Overall, using the data generated with this approach, we determined to what extent (i) cell type-specific differences in metabolic fluxes between porcine PBMCs and granulocytes and (ii) sex-specific differences in metabolic patterns of the two cell types become apparent. 

## 2. Materials and Methods

### 2.1. Isolation of Granulocytes and PBMCs from Whole Blood

The study was approved by the University of Ulm Animal Care Committee and the Federal Authorities for Animal Research (Regierungspräsidium Tübingen; Reg.-Nr. 1559, approval 29 October 2021) and is in compliance with the National Institute of Health Guidelines on the Use of Laboratory Animals and the European Union “Directive 2010/63/EU on the protection of animals used for scientific purposes”. According to the 3R principle (replacement, reduction, and refinement in animal research), the present study is a post hoc analysis of material available from animal experiments recently described by Münz et al. [35]. A total of 12 healthy, human-sized, sexually mature German landrace pigs (range body weight 63–102 kg) of either sex (each *n* = 6) between the ages of 21 and 27 weeks had been included in that study. Blood collection under anesthesia and cell purification of the PBMCs and granulocytes using Ficoll density centrifugation were performed as described in detail by Münz et al. [35].

### 2.2. Measurement of Immune Cell Superoxide Anion Production and Mitochondrial Respiration

Superoxide anion radical (O_2_^•−^) production rate and mitochondrial O_2_ fluxes (JO_2_) of ROUTINE respiration of the purified PBMCs and granulocytes were analyzed with a EMXnano electron spin resonance (ESR) spectrometer (Bruker, Billerica, MA, USA) and high-resolution respirometry using an Oroboros^®^ Oxygraph-2K (Oroboros Instruments, Innsbruck, Austria), as described in the original publication [35].

### 2.3. Ex Vivo ^13^C-Tracer Experiments of PBMCs and Granulocytes

For the parallel isotopic labeling experiments, 5 × 10^6^ purified granulocytes and PBMCs were resuspended in 1 mL RPMI medium (Genaxxon bioscience, Ulm, Germany, detailed composition is listed in the Supplementary Material) spiked with an isotopic tracer (all from Cambridge Isotope Laboratories, Andover, MA, USA) containing [1,2-^13^C]glucose, [4,5,6-^13^C]glucose, [U-^13^C]glucose (each labeled/unlabeled, 1:1, *w*/*w*) or [U-^13^C]glutamine (100% labeled), respectively (*n* = 2, for each ^13^C-tracer experiment). The incubation was performed in a closed 2 mL tube at 37 °C under constant vertical rotation at 5 rpm (Trayster Digital, IKA, Staufen, Germany) for 2 h. After incubation, the cells were spun down and 850 μL of the supernatant were transferred into a vial and subsequently stored at −20 °C until the GC-MS analysis of the ^13^CO_2_ enrichment and lactate. The cell pellet was washed once with a 0.9% NaCl solution and subsequently stored at −80 °C after removal of all the liquid. The samples were stored for about 2 weeks until analysis.

### 2.4. Extraction of Intracellular Metabolites

The water/methanol–acetonitrile extraction was performed as recently described in Hogg et al. [33]. Briefly, the frozen pellet was extracted with 100 μL ice-cold Ampuwa water, vortexed vigorously, and sonicated in an ice bath for 10 min. Next, 400 μL of cold methanol/acetonitrile (1:1, *v*/*v*, −20 °C) were added to the suspension. After extraction for 10 min using an ultrasonic cleaning bath, the samples were spun down at 14,000 g and 4 °C for 5 min. The supernatant from duplicates of the isotopic tracer experiments were pooled in a 1.5 mL vial. The solvent was removed by a SpeedVac evaporator (Savant SPD2010 SpeedVac concentrator, Thermo Scientific, Waltham, MA, USA) at 45 °C, and the residue was kept overnight at −20 °C until derivatization and GC–MS analysis.

### 2.5. Derivatization and GC-MS Analysis of Intracellular Metabolites

The ethoxime-trimethylsilyl derivatization (EtOx-TMS) and following GC-MS analysis were performed as recently described [33]. Briefly, the dried cell extracts and at least five staggered levels of reference standard mix containing all the monitored metabolites (∑19; list is available in the Supplementary Material) were dissolved in 50 μL derivatization reagent (ethoxyamine hydrochloride in pyridine (2%, *w*/*v*) (Sigma-Aldrich, St. Louis, MO, USA) and sonicated for 10 min at room temperature. The derivatization was performed at 60 °C for 60 min. The samples were evaporated to dryness with a gentle stream of nitrogen at 45 °C and redissolved in 30 μL acetonitrile and 30 μL BSTFA (N,O-bis(trimethylsilyl)trifluoroacetamide) (abcr, Karlsruhe, Germany) by ultrasonication for 15 min. For derivatization, the reaction mixture was heated to 60 °C for 45 min. The sample was centrifuged for 5 min at 14,000× *g*, and the clear liquid was transferred into a GC vial for GC-MS analysis. All the samples were analyzed twice on a 7890A/5977B GC-MS system (Agilent, Waldbronn, Germany) equipped with a 30 m OPTIMA^®^ 1301-MS column (6% cyanopropylphenyl-/94% dimethylpolysiloxane, 0.25 mm internal diameter, 0.25 μm film thickness; Macherey-Nagel, Düren, Germany). The GC-electron ionization (EI)/MS data were collected in the selected ion monitoring (SIM) mode. Further details about the SIM parameters and GC-MS settings are provided in the Supplementary Material.

### 2.6. GC-MS Analysis of ^13^CO_2_ Enrichment

After thawing of the frozen supernatant, 25 μL 1N HCl was injected into the septum-capped vial, and the vial was vigorously shaken to release the CO_2_. The liquid was spun down, and the CO_2_ in the headspace was analyzed by a 7890B/5975C MSD GC-MS system (Agilent, Waldbronn, Germany), which was equipped with a 30 m OPTIMA^®^ 5-MS column (95% methyl- and 5% phenyl polysiloxane, 0.25 mm internal diameter, 0.25 μm film thickness; Macherey-Nagel, Düren, Germany). 5 μL headspace gas was injected 10 times in split mode (20:1) at a constant oven temperature of 100 °C. The total run time was 5 min. All the measurements were performed in the SIM mode, including *m*/*z* 44 and *m*/*z* 45. The isotopic enrichment tracer-to-tracee ratio (TTR) (Equation (1)) was determined for the calculation of the total secreted ^13^CO_2_ production (Equation (2)):

(1) TTR=CO132CO122sample−CO132CO122background

(2) CO132 production nmol1×106cells=TTR×E μmcell density 106cellsmL

where E is the concentration of sodium bicarbonate in the RPMI medium.

### 2.7. Derivatization and GC-MS Analysis of Secreted Lactate

For the quantification of lactate released into the medium, a total of eight lactate standards in a concentration range of 0/0.01 to 0.5 μM were prepared in the RPMI medium. After the GC-MS analysis of CO_2_, 25 μL of internal standard (IS) (0.5 μM 3-hydroxy-propanoic acid solution in water, Sigma-Aldrich, St. Louis, MO, USA) were spiked with 100 μL of the supernatant or 100 μL of standard solution. The sample was evaporated in a SpeedVac at 45 °C. Subsequently, the residue was resuspended in 600 μL acetonitrile and 25 μL MTBSTFA (N-methyl-N-tert-butyldimethylsilyltrifluoracetamid) (abcr, Karslruhe, Germany). The vial was tightly sealed and incubated at 80 °C for 1 h before being subjected to GC-MS analysis. A sample volume of 1 μL was injected in duplicate into a HP 6890 GC/5973 MSD system (Hewlett-Packard/Agilent, Waldbronn, Germany) equipped with a 12 m OPTIMA^®^ 5-MS column (0.2 mm internal diameter, 0.35 μm film thickness; Macherey-Nagel, Düren, Germany). The GC oven program and GC-MS temperatures are listed in the Supplementary Material. The characteristic fragment ion [M-57]^+^ of the lactate and IS (*m*/*z* 261.1–266.1, C1–C3) were analyzed in SIM mode.

### 2.8. Metabolic Flux Analysis

**Figure 1:**
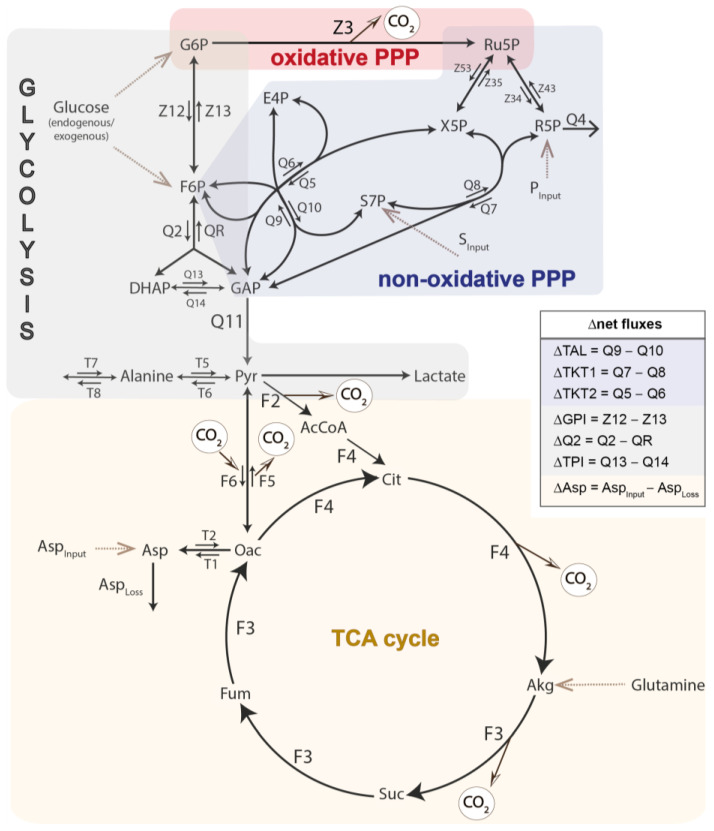
Simplified metabolic network of PBMCs and granulocytes. Each arrow indicates a to-be-calculated flux in the model. Dotted arrows mark substrate input fluxes.

For ^13^C-MFA, the measured mass isotopomer distribution was corrected for the presence of naturally occurring isotopes other than carbons, resulting in the carbon mass distribution (CMD) [36]. The metabolic network included all the major pathways of the central carbon metabolism: PPP, glycolysis, gluconeogenesis, and the TCA cycle (Figure 1). Net-fluxes of reversible reactions were denoted by the symbol “Δ”, and definitions are shown in Figure 1. ^13^C-MFA was performed with our previously established Bayesian ^13^C-MFA routine using the rstan package v2.21.2 (R interface to Stan) [33,34]. In brief, we computed the CMDs for the given fluxes based on the elementary metabolite unit (EMU) approach and compared them with the corresponding GC-MS measurements. If the calculated CMDs were comparable to the GC-MS measurements, the sample was collected in a Markov chain Monte Carlo (MCMC) sampling chain; otherwise, it was discarded. The MCMC algorithm was used to determine the precise flux distributions with their joint confidence intervals. The absolute flux rates were calculated based on the determined ^13^CO_2_ production and lactate accumulation in the medium.

### 2.9. Measurement of Circulating Steroid Levels and Cytokine Plasma Levels

As described previously [35], testosterone and aldosterone were determined using commercially available ELISA kits for pig plasma, according to the manufacturer’s instructions (Abnova, Taipei City, Taiwan/BlueGene Biotech, Shanghai, China). 17-β-estradiol (E2) in the serum was determined by the central laboratory for Clinical Chemistry of the University Hospital in Ulm (Germany) using electrochemiluminescence immunoassay technology with a Cobas e801 immunoassay analyzer (Roche Diagnostics, Mannheim, Germany).

### 2.10. Flow Cytometry

The antibodies used and the formulation of the fluorescence-activated cell sorting (FACS) buffer are listed in the Supplementary Material. The purified PBMCs were resuspended in FACS buffer +10% pig serum (Pan Biotech, Aidenbach, Germany). The primary antibodies (CD3), as well as the directly labeled antibodies (CD4, CD8) and their isocontrols were added to the respective tubes, vortexed shortly, and incubated for at least 20 min on ice. The cells were washed once with 1 mL FACS buffer. The cell pellets were resuspended in 100 μL FACS buffer +10% pig serum, and the secondary antibodies (APC IgG1) were added and incubated for at least 10 min on ice. After incubation, the cells were washed once with FACS buffer and then resuspended in 200 μL FACS buffer. The samples were transferred to a 96-well plate and analyzed by flow cytometry (Beckman Coulter Cytoflex, Brea, CA, USA). Data acquisition and analysis were performed using CytExpert 2.4 software (Beckman Coulter Life Sciences, Brea, CA, USA).

### 2.11. Statistical Analysis

The means of the technical replicates were used for statistical analysis, i.e., reported flux values reflected the mean of the posterior distribution. For facilitated group comparison, the medians with the interquartile range (IQR) were presented as error bars in the graphs. The Mann–Whitney U test was used to compare group differences with differences considered significant when *p* < 0.05. The normal distribution was tested with the Shapiro–Wilk test. If the data were not normally distributed, linear correlation coefficients were calculated according to Spearman; otherwise, the Pearson correlation coefficient was the preferred method for normally distributed data. The statistical analyses mentioned above were conducted using GraphPad Prism software (version 10.0.2). The PCA was performed with analyzed fluxes of the PBMC and granulocyte metabolism (each *n* = 12), including mitochondrial O_2_ consumption, glycolytic fluxes (hexose (glucose/fructose) uptake rate, Z3, ΔTAL, ΔTKT1, input S7P, Q4, ΔQ2, Q11, lactate production rate), TCA cycle fluxes (F2, F3, F4, F6 glutamine uptake rate, ΔAsp), and O_2_^•−^ production (flux definitions presented in Figure 1). The input data were standardized to a sample mean of zero and a unit sample standard deviation of 1 before performing the PCA in RStudio (version 2022.12.0) with the following built-in R functions: prcomp, varimax (package stats), and pracma [37,38]. We used the jackknife test, also called the “leave one out” procedure, for cross-validation [39]. The parameters (fluxes) were considered significant when the relative error (jackknife-SE over nominal flux value) was smaller than 0.5.

## 3. Results

### 3.1. ^13^C-MFA of Granulocytes and PBMCs: Sex-Specific Differences in the Pentose Phosphate Pathway

**Figure 2:**
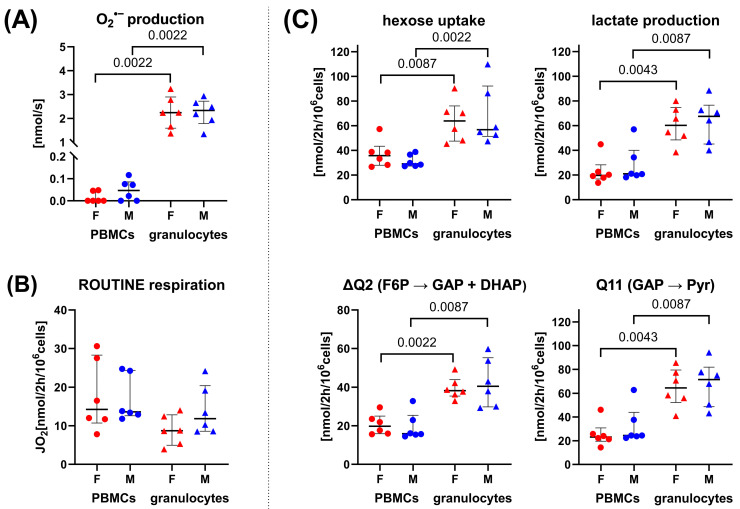
O_2_^•−^ production, ROUTINE respiration, and glycolytic fluxes of circulating immune cells. Individual data of PBMCs are displayed as circles and granulocytes as triangles, whereby immune cell data of females are highlighted in red and males in blue. Error bars show the median with IQR of each group (*n* = 6). *p* values were determined with the Mann–Whitney U test. (**A**) O_2_^•−^ production rate as determined by electron spin resonance spectrometry; values below the RPMI-blank value were set to zero (*n* = 4 for PBMCs of females; *n* = 2 for males). (**B**) ROUTINE respiration (physiological coupling state) as determined by high resolution respirometry. (**C**) Absolute fluxes of the glycolytic pathway. Individual dots indicate the posterior mean from Bayesian ^13^C-MFA.

**Figure 3:**
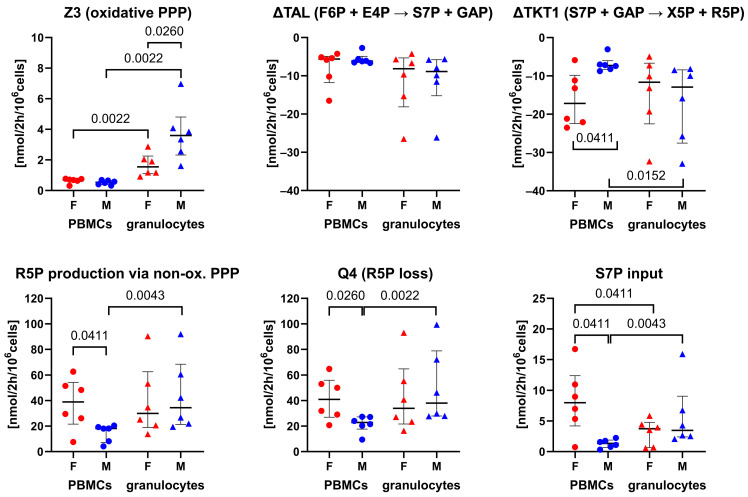
^13^C-MFA results of the oxidative and non-oxidative pentose phosphate pathway. Individual data of PBMCs are displayed as circles and granulocytes as triangles, whereby immune cell data of females are highlighted in red and males in blue. Error bars show the median with IQR of each group (*n* = 6). *p* values were determined with the Mann–Whitney U test. Individual dots indicate the posterior mean from Bayesian ^13^C-MFA. Negative net fluxes (ΔTAL, ΔTKT1) indicate a shift of the non-oxidative PPP fluxes toward ribose-5-phosphate production.

**Figure 4:**
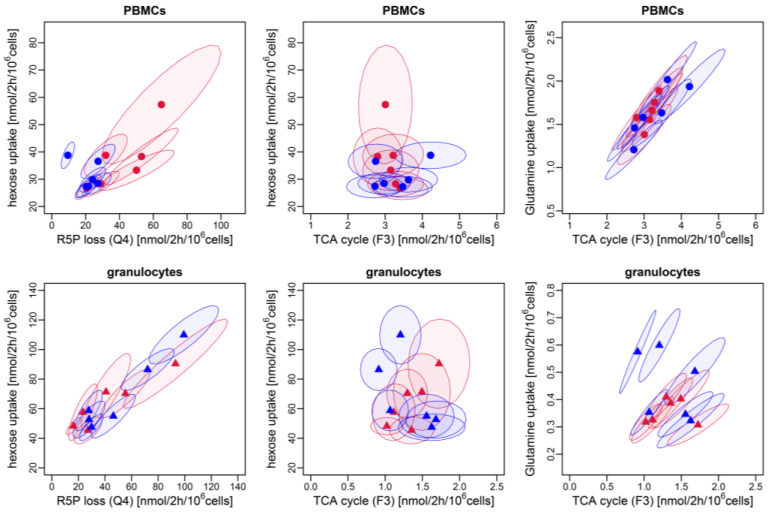
Flux correlations in PBMCs and granulocytes. (**Left**): Relations between hexose (glucose/fructose) uptake rate and R5P loss (Q4). (**Middle**): Relations between hexose (glucose/fructose) uptake rate and the TCA cycle flux F3 (Akg → Oac + CO_2_). (**Right**): Relations between glutamine uptake into the TCA cycle and the TCA cycle flux F3 (Akg → Oac + CO_2_). Bayesian ^13^C-MFA results are displayed as an ellipse with 68% confidence interval (±one standard deviation of the mean) of posterior distributions with posterior mean indicated by symbols (PBMCs: circles, granulocytes: triangles). Data from female pigs are highlighted in red and males in blue.

**Figure 5:**
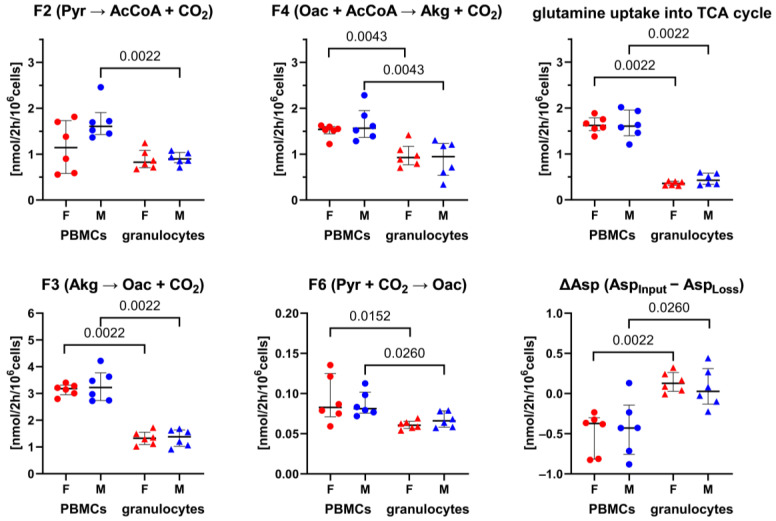
^13^C-MFA results of TCA cycle fluxes including glutamine utilization and net loss of aspartate. Individual data of PBMCs are displayed as circles and granulocytes as triangles, whereby immune cell data of females are highlighted in red and males in blue. Error bars show the median with IQR of each group (*n* = 6). *p* values were determined with the Mann-Whitney-U test. Individual dots indicate the posterior mean from Bayesian ^13^C-MFA. The negative net flux (ΔAsp) indicates the release of aspartate for protein and nucleotide biosynthesis.

Glycolytic fluxes including hexose (glucose/fructose) uptake rate, glycolytic triose formation (ΔQ2: F6P → GAP + DHAP), glyceraldehyde-3-phophate (GAP)-derived pyruvate production (Q11: GAP → Pyr), and lactate production were at least two-fold higher in the granulocytes than in the PBMCs (Figure 2). The difference between the two cell types was most pronounced for Q11 (GAP → Pyr) and lactate production, indicating that the PBMCs utilized trioses mainly via the non-oxidative PPP to generate R5P, whereas the granulocytes converted trioses to lactate. For both cell types, there were no intergroup differences between the sexes in any of the observed glycolytic fluxes (Figure 2). The oxidative PPP (Z3) was significantly lower in the PBMCs (range 0.3 to 0.8 nmol/2 h/10^6^ cells) when compared to the granulocytes (range 0.9–7.0 nmol/2 h/10^6^ cells). While the PBMCs showed no sex-differences in the oxidative PPP (Z3), in the granulocytes, this flux was about two-fold higher in males than in females (Figure 3). However, the higher net reverse flux of transketolase 1 (∆TKT1: S7P + GAP → X5P + R5P), together with higher rates of incorporation of sedoheptulose-7-phosphate (S7P) into the non-oxidative PPP led to significantly higher R5P production rates in the PBMCs of females compared to males. R5P loss (Q4) of the granulocytes of both sexes and the PBMCs of females was comparable, while the male PBMCs generated lower amounts of R5P via the non-oxidative PPP to sustain biosynthetic processes. Interestingly, we observed a strong linear correlation between the hexose (glucose/fructose) uptake rate and R5P loss (Q4) for the biosynthetic processes in the granulocytes (Pearson correlation coefficient r = 0.939, *p* < 0.0001 (*n* = 12)), while no correlation was present for the PBMCs (Figure 4). A small fraction of the glucose-derived pyruvate was transformed into acetyl-CoA that feeds the TCA cycle (F2), whereby the proportion was greater in the PBMCs (median 5.95, IQ: 3.3) compared to the granulocytes (median 1.35, IQ: 0.6). We observed no sex-related differences within the PBMCs or granulocytes for any TCA cycle fluxes (F3, F4) or for the pyruvate-derived fraction of acetyl-CoA (F2), glutamine uptake, and net aspartate loss (Figure 5). However, the TCA cycle activity of the PBMCs was significantly higher compared to the granulocytes, with the greatest differences observed in the glutamine uptake and its metabolism via the TCA cycle to four-carbon metabolites, like oxaloacetate/aspartate (F3). Interestingly, the glutamine uptake rate significantly correlated with these TCA cycle fluxes in the PBMCs (Pearson correlation coefficient r = 0.815, *p* = 0.0013 (*n* = 12))*,* while no relation was found in the granulocytes. For both cell types, no relations were found between the hexose (fructose/glucose) uptake rate and the TCA cycle fluxes (F3: Akg → Oac + CO_2_, F4: Oac + AcCoA → Akg + CO_2_) (Figure 4; Figure S1).

### 3.2. Theoretical Calculation of ATP Generation Rates Based on ^13^C-MFA Results

The absolute quantification of the fluxes enables the theoretical calculation of the maximum ATP yields of the two main ATP-producing pathways: glycolysis vs. TCA cycle-fueled oxidative phosphorylation (Table 1). The PBMCs primarily used the TCA cycle to meet their energy demand, while the granulocytes mainly relied on glycolysis to generate ATP. Independently of cell type or sex, the spread of data was greater in the glycolysis-derived ATP production than in the TCA cycle. This can be attributed to the fact that the TCA cycle is a “closed” system, while glycolysis and the PPP compete for G6P to produce ATP or R5P for biosynthesis, respectively. Hence, R5P production decreased the ATP production rate via glycolysis, which could explain the data variance. The total ATP production of both cell types was comparable, and we observed no sex-related differences in the ATP production rate of both cell types.

### 3.3. Identification of Metabolic Patterns with PCA: Granulocytes and PBMCs Differ in Their Metabolic Profile

**Figure 6:**
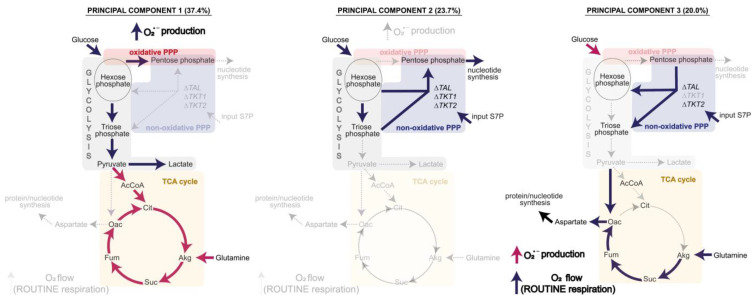
Identification of metabolic patterns in the three principal components (PC) of the PCA. Contribution of each PC to the total variance is presented within parenthesis. Dark blue arrows indicate the positively linked pathways and red arrows show the corresponding negatively linked pathways. Dotted, faded arrows indicate fluxes that have no significant contribution to the respective PC.

**Figure 7:**
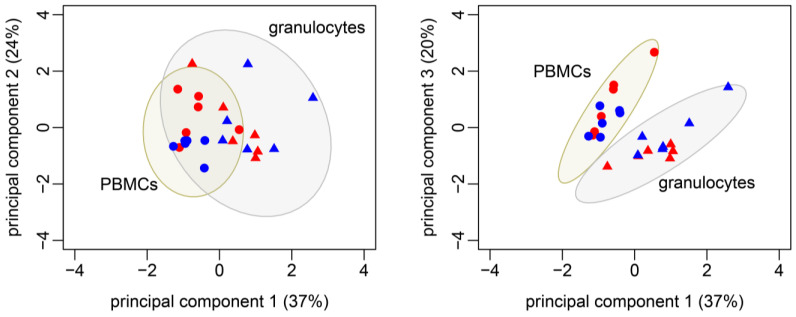
PCA biplots based on metabolic fluxes, O_2_^•−^ production, and oxygen consumption of 24 datasets. Contribution of each PC to the total variance is indicated within parenthesis. The 95% confidence ellipse is shown for granulocytes (triangles, *n* = 12) and PBMCs (circles, *n* = 12) each. Circulating immune cells from female pigs are highlighted in red and males in blue.

We further evaluated the interrelationships among the fluxes by subjecting the obtained dataset to a PCA. The dimensionality of the datasets was reduced by identifying three linear combinations of variables (called principal components (PC)) that explained 81% of the data variance. The PC1 shows a coupling of glycolysis, oxidative PPP (Z3), and O_2_^•−^ production in the positive direction, while the glutamine uptake, pyruvate transformation into acetyl-CoA (F2: Pyr → AcCoA +CO_2_), and TCA cycle fluxes (F3: Akg → Oac + CO_2_, F4: Oac + AcCoA → Akg + CO_2_) were negatively linked (Figure 6; Table 2). Thus, the TCA cycle fluxes and glucose catabolism can be clearly separated along the PC1 axis, which accounts for more than one-third of the total variance of the flux data. PC2 refers to the anabolic glucose metabolism through the non-oxidative PPP, while PC3 mainly considers glutamine utilization via the TCA cycle to promote biosynthetic processes. The latter pathway was positively linked with ROUTINE respiration and negatively linked with radical production. The resulting score plot between PC1 and PC3 allows a clear separation between the cell types, whereby separation was greater along PC1 (Figure 7). However, the 2D-PCA plots revealed no sex-specific clusters.

### 3.4. Average Percentage of T Helper Cells (CD3^+^CD4^+^) in PBMCs Was Higher in Females Than Males

**Figure 8:**
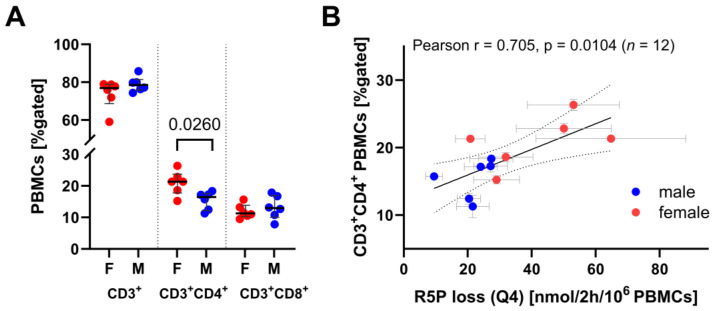
(**A**) Average values of PBMC subsets from porcine blood as determined by flow cytometry (*n* = 3, technical replicates). Error graphs show the median with IQR of each group (*n* = 6), whereby immune cell data from females are highlighted in red and males in blue. *p* values were determined with the Mann–Whitney U test. (**B**) Linear regression between pentose loss (Q4) and CD3^+^CD4^+^ subset of PBMCs (R^2^ = 0.4975). The dashed lines indicate the 95% confidence interval. R5P loss was determined by Bayesian ^13^C-MFA. Error bars indicate the standard deviation of the posterior distribution. We found no statistically significant correlation within a sex-specific group with *n* = 6.

The average percentage of T helper cells (CD3^+^CD4^+^) was higher in the females compared to the males, while the percentages of total T cells (CD3^+^) and cytotoxic T (CD3^+^CD8^+^) cells were comparable between both sexes (Figure 8A). The frequency of the T helper cell subset closely correlated with the flux of the R5P loss in the PBMCs (Figure 8B).

### 3.5. Sex-Specific Differences of Circulating Steroid Hormone Levels and Their Association with Metabolic Fluxes of Immune Cells

**Figure 9:**
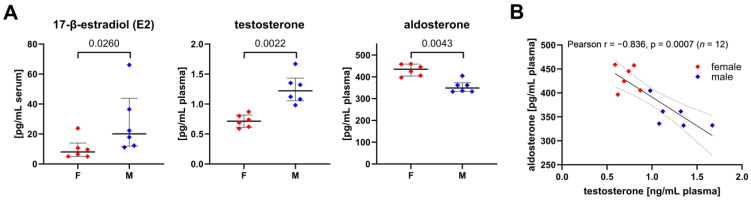
Sex steroid plasma/serum levels and corresponding correlations. Aldosterone and testosterone were determined by ELISA, while 17-β-Estradiol was measured by ECLIA. (**A**) 17-β-estradiol, aldosterone, and testosterone levels in serum/plasma. Error bars show the median with IQR of each group (*n* = 6), whereby samples collected from female pigs are indicated as red diamonds and males as blue diamonds. (**B**) Linear regression between aldosterone and testosterone plasma levels (R^2^ = 0.6986). The dashed lines indicate the 95% confidence interval. We found no statistically significant correlation within a sex-specific group with *n* = 6.

**Figure 10:**
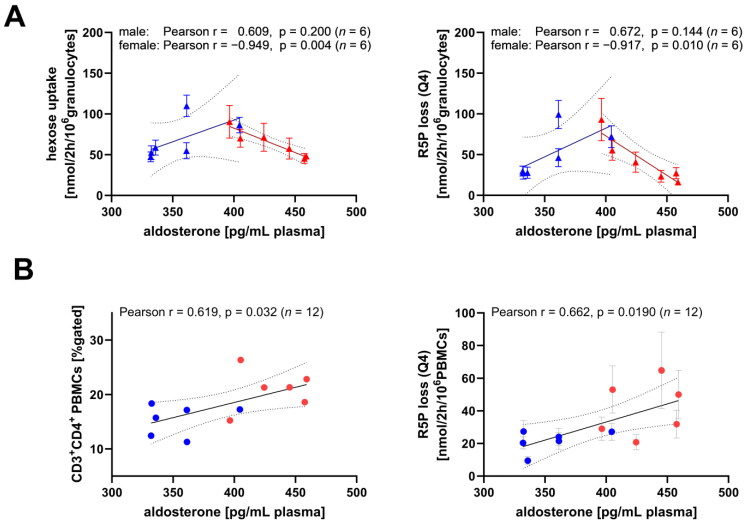
Relation between aldosterone plasma levels and glycolytic/PPP fluxes of circulating immune cells. Data from males are depicted as blue symbols and data from females as red symbols. (**A**) Granulocytes (triangles, *n* = 6, each sex), (**B**) PBMCs (circles, *n* =12 of both sexes). Metabolic fluxes were determined by our ^13^C-MFA approach, where posterior mean was used for evaluation. CD3^+^CD4^+^ subpopulation of PBMCs were analyzed by flow cytometry. For PBMCs, we found no statistically significant correlation within a sex-specific group with *n* = 6. Lines represent the linear regression fit, and the dashed lines indicate a 95% confidence interval.

The testosterone plasma levels were about 1.7-fold higher in the males than the females (median (IQR) (male: 1.20 (0.25) mg/mL, female: 0.72 (0.18) mg/mL)), while the 17-β-estradiol (E2) serum levels were over 2.5-fold higher in the males compared to the females (male: 20 (19) ng/mL, female: 8 (5) ng/mL) (Figure 9A). The spread of 17-β-estradiol levels within each group included biological variance induced by estrus cycles. The determined estradiol levels of the female pigs ranged from 5 to 23.8 pg/mL (*n* = 6), which align with the literature reports of Soede et al. showing that porcine 17-β-estradiol levels range from baseline (zero) to approx. 23 pg/mL during the estradiol cycle [41]. We did not observe any correlation between sex steroid hormones and metabolic fluxes of the PBMCs and granulocytes but found an inverse relation between the aldosterone and testosterone plasma levels (Figure 9B). The females had significantly higher aldosterone plasma concentrations than the males (males: 349 (28) pg/mL, females: 435 (44) pg/mL). Furthermore, we investigated whether the aldosterone plasma levels were related to immune cell metabolic fluxes and whether these relations differed between the sexes (Table 3, Figure 10). Both the R5P loss via the non-oxidative PPP in the PMBCs and the CD3^+^CD4^+^ subset of the PBMCs showed a strong direct correlation with the aldosterone plasma levels (Figure 10B). The hexose (glucose/fructose) uptake rate in the granulocytes from the females was directly correlated with the aldosterone plasma levels, whereas we observed an inverse correlation for the males (Table 3). Furthermore, the strength of glucose anabolism via the non-oxidative PPP to promote R5P production was negatively associated with the aldosterone levels, whereby this correlation was only present in the granulocytes from the females. Interestingly, we observed a trend towards a reverse relationship in the granulocytes from the males but found no statistically significant correlations. These sex-specific differences that were found in the relation of the aldosterone plasma levels with non-oxidative PPP fluxes in the granulocytes align with the second principal component of our PCA (hexose (glucose/fructose) uptake, ΔTAL, ΔTKT1, S7P input, and R5P loss (Q4)) (Table 2 and Table 3).

## 4. Discussion

We investigated ex vivo the immunometabolism of the granulocytes and PBMCs. ^13^C-MFA detected sex-specific differences in the PPP. The PBMCs of the females showed significantly higher ribose-5-phosphate production via the non-oxidative PPP, while the granulocytes of the males had higher oxidative PPP activity. Furthermore, by combining data from the ^13^C-MFA of the glycolysis/PPP and TCA cycle, as well as the O_2_^•−^ production and mitochondrial respiration, we could identify cell type-specific patterns of metabolic plasticity that acted independently of sex.

The PBMCs displayed higher TCA cycle activity compared to the granulocytes, especially glutamine-derived aspartate biosynthesis, which was associated with mitochondrial respiration and negatively linked to O_2_^•−^ production. In contrast, the granulocytes mainly relied on glucose to meet their energy demand (~79% of the whole ATP production), which was coupled with oxidative PPP utilization and O_2_^•−^ production rates. The non-oxidative PPP explained almost a quarter of the variation in all the flux observations (PC2; ~24%) and did not allow a separation between the PBMCs and granulocytes, indicating that both cell types have a high non-oxidative PPP flux rate to supply R5P for nucleotide synthesis. As granulocytes are characterized by a low proliferative capacity and a short life cycle (< 1 day), there was a surprisingly high demand for R5P that is typically elevated during DNA synthesis processes. One possibility is the requirement of R5P for the synthesis of microRNAs, which regulate the gene expression of neutrophils and thus control the homeostasis in health and disease [42]. Another possibility is that the non-oxidative PPP enhanced R5P synthesis for mRNA expression. For example, the mRNA of matrix metalloproteases (MMP-9) or (pro)angiogenic molecules (VEGF-A) was significantly increased in the neutrophils upon aldosterone stimulation at physiological concentrations (10^−8^ M) and mediated by mineralocorticoid receptors (MR) [43,44]. Overall, these findings indicate a thus far underestimated importance of nucleotide synthesis in granulocytes.

In most previous studies investigating immune cell metabolism, sex was not considered as a biological variable. However, our results suggest sex-specific and aldosterone-induced effects of the R5P production in neutrophils, since we found an inverse correlation between aldosterone and non-oxidative PPP fluxes in the granulocytes from the females, while the males showed a trend towards the opposite pattern. Furthermore, sex-specific metabolic differences in human neutrophils were recently reported by Gupta et al., who measured respiration (OCR) and the extracellular acidification rate (ECAR) to estimate glycolysis and found significantly higher mitochondrial respiration in the neutrophils of males. This was the case for both the absolute values of mitochondrial respiration, as well as values in relation to glycolysis (OCR/ECAR), while the glycolytic rate alone did not differ (*n* = 6 per sex) [45]. The authors linked these differences to estradiol, as values of mitochondrial metabolism became comparable to untreated females once the neutrophils from males were incubated with estradiol.

While our data aligns with Gupta et al. regarding the absence of sex-specific particularities in glycolysis, their finding of increased mitochondrial metabolism in males was not reflected in our OCR measurements or in the quantitatively determined TCA cycle fluxes. Interestingly, the male pigs had significantly higher estradiol serum levels than the females in our study, which is consistent with findings from Clapper et al., who had reported on this peculiarity of sex hormones in boars [46]. These higher estradiol serum levels in male pigs compared to females may have disguised the sex-specific increase in OCR observed by Gupta et al. [45]. In return, sex-specific differences were found in the oxidative PPP, as the flux was significantly increased in the granulocytes from males. This is particularly interesting, considering the importance of the PPP for neutrophil function. Correspondingly, we expected a link between the oxidative PPP and O_2_^•−^ production in neutrophils, since the oxidative PPP is the main source of NADPH for the NADPH oxidase, an enzyme that catalyzes the electron transfer from NADPH to molecular oxygen and reduces the latter to O_2_^•−^ [47]. Furthermore, the results of our PCA support our hypothesis and indicate that the oxidative PPP is the main metabolic pathway for O_2_^•−^ production in granulocytes. However, we did not observe the same sex-specific difference in the O_2_^•−^ production as in the oxidative PPP. Brown et al. reported similar results when analyzing the ROS generation of non-stressed neutrophils isolated from the peripheral blood of rats of both sexes [48]. We speculate that the increased oxidative PPP combined with unchanged O_2_^•−^ production in the granulocytes of males may be a consequence of the antioxidative properties of sex steroids. Several studies have shown that stimulation of granulocytes/neutrophils with sex steroids (e.g., estrogen and testosterone) in physiological concentrations (10^−8^ M) significantly reduces O_2_^•−^ production rates [5,22,23]. Our male pigs displayed significantly higher testosterone and estradiol plasma/serum concentrations than the females, although these levels were still low compared to the ones typically used for in vitro stimulation experiments of granulocytes. Interestingly, Marin et al. pointed out that the radical scavenger capacity of testosterone is higher at physiological levels than at higher concentrations [23]; thus, it is tempting to speculate that the increased oxidative PPP in male granulocytes is not reflected in the O_2_^•−^ production due to the potential antioxidative effects of estradiol and testosterone. Furthermore, the increased NADPH release via oxidative PPP may promote fatty acid synthesis without affecting the O_2_^•−^ production.

Similar to granulocytes, we observed sex-specific differences in the PPP fluxes in the PBMCs: the PBMCs of the females released higher amounts of R5P via the non-oxidative PPP compared to the males. Glycolysis and the TCA cycle remained unaffected. Furthermore, in line with the reports of Abdullah et al., who reported a similar effect in humans, we showed that the porcine lymphocytes of the females had a significantly higher abundance of T helper cells (CD3^+^CD4^+^) compared to the males [49]. The strong correlation in the PBMCs between the T helper population and the non-oxidative PPP-mediated R5P loss leads us to speculate that the non-oxidative PPP regulates the homeostasis of CD3^+^CD4^+^ T helper cells. This effect was not associated with sex steroid hormone plasma, but with aldosterone levels. Aldosterone is a mineralocorticoid hormone that plays a central role in the homeostatic regulation of blood pressure, but is also now widely accepted for activating cells in the innate and adaptive immune system, which is associated with metabolic and cardiovascular diseases [50]. Despite the findings that PBMCs express mRNA of MR [51], little is known about the processes triggered by MR/aldosterone binding. The aldosterone plasma concentration in the female pigs was significantly higher compared to the males, analogously to reports in humans by other groups [52,53]. Furthermore, we observed a significant inverse linear correlation between the aldosterone and testosterone levels, which agrees with a previous study showing that testosterone can inhibit the aldosterone production in male rats [54]. Therefore, it seems likely that the sex-related differences were indirectly driven by testosterone. 

Silaidos et al. reported further sex-associated differences in human PBMCs from healthy volunteers of reproductive age regarding their mitochondrial respiration and citrate synthase activity, with both parameters being significantly higher in females compared to males [9]. In addition, it has been shown that the mitochondrial ROS production was reduced in females compared to males due to the presence of estrogens [55]. In contrast to human volunteer studies, we did not find any sex-associated differences in mitochondrial respiration, the TCA cycle fluxes, or O_2_^•−^ production in the PBMCs from sexually mature pigs. This species-specific metabolic behavior may be related to the presence of estrogens, e.g., 17-β-estradiol (E2). Our determined estradiol levels in the male and female pigs were in good agreement with reports from Jayachandran et al. [56], which have also shown that changes in E2 serum levels from juvenile to adult pigs were not significantly different between sexes [56]. In contrast, E2 levels in healthy women increase during puberty and peak at reproductive age (65–125 pg/mL) [57,58]. Thus, E2 levels are dramatically lower in adult female pigs compared to pre-menopausal women. Due to species-specific levels of sex steroids, estrogen-related effects observed in adult humans may not be mirrored in the porcine model. However, the PC3 of our PCA confirmed the link between mitochondrial respiration and ROS production: high glutamine-derived aspartate biosynthesis via the TCA cycle and high oxygen consumption rates were associated with decreased O_2_^•−^ production, which is in line with previous reports demonstrating that increased electron transport reduced ROS production [59]. In conclusion, the PC3 of PCA provided an explanation for the interplay between the higher mitochondrial activity in females, as reported by Silaidos et al. [9] and the positive estrogen-associated effects on mitochondrial stress levels and ROS formation, as demonstrated by others [55,59].

It is noteworthy that all the effects described in this study refer to the resting state of granulocytes and PBMCs. Therefore, we cannot draw conclusions about the extent to which these basal effects impact immune cells during inflammation. It is well documented that aldosterone can lead to increased polarization of CD4^+^ cells into proinflammatory subsets (Th17, Th1) and that the following aldosterone-mediated immune activation can play an elevated role in some diseases, e.g., autoimmunity and metabolic syndrome [50,60,61]. Furthermore, it has been demonstrated that disease progression in men and women can differ dramatically due to sex [62,63,64]. Thus, the determined (sex-specific) correlations between T cell subsets (CD3^+^CD4^+^), aldosterone plasma levels, and the PPP activity in circulating immune cells (granulocytes and PBMCs) may provide valuable information that contributes to the elucidation of the exact molecular mechanisms and may assume importance in the progression and/or prevention of diseases.

### Limitations and Perspectives

To minimize animal experiments in accordance with the 3R principle, the blood samples required for this work were collected as part of our recently published pilot study involving anesthetized and mechanically ventilated healthy pigs. For this pilot experiment, we were only able to obtain permission for 12 animals from the Animal Care Committee of the University Ulm and the Federal Authorities for Animal Research (Regierungspräsidium Tübingen). Conclusively, a power calculation had been impossible. A drawback of ^13^C-MFA is the number of required cells for parallel tracer experiments with ^13^C-labeled substrates; therefore, additional stimulation experiments were not possible in this exploratory study due to the limited blood volume obtained from the animals. Based on the complex nature of the ex vivo analysis, we cannot prove whether sex differences were directly influenced by sex hormones or indirectly by the interaction of multiple factors (e.g., interplay between sex steroids and aldosterone). This calls for targeted in vitro stimulation experiments and additional studies to elucidate the underlying mechanisms. Furthermore, sex-related effects of the PPP activity, a key regulator of cellular mechanisms (e.g., proliferation and survival), may provide specific therapeutic options in a variety of diseases. We therefore emphasize the importance of considering sex as a biological variable when designing studies investigating immunometabolism in health and disease.

## 5. Conclusions

In this study, we determined cell type-specific differences in the metabolic patterns between PBMCs and granulocytes. The PBMCs displayed higher TCA cycle utilization, especially glutamine-derived aspartate biosynthesis, which was associated with mitochondrial respiration and negatively linked with O_2_^•−^ production. In contrast, the granulocytes mainly utilized glucose via anaerobic glycolysis, which was coupled with oxidative PPP utilization and O_2_^•−^ production rates. Both cell types showed a high non-oxidative PPP flux rate to supply R5P for nucleotide synthesis. Regarding the sex-specific differences in immunometabolism, the granulocytes of males had higher oxidative PPP fluxes compared to females. In contrast, the PBMCs of females displayed higher non-oxidative PPP fluxes compared to males, associated with the T helper cell (CD3^+^CD4^+^) subpopulation of PBMCs. The observed sex-specific differences were not directly attributable to sex steroid plasma levels, but we detected an inverse correlation between testosterone and aldosterone plasma levels and showed that aldosterone levels were related to non-oxidative PPP fluxes of both cell types.

## Figures and Tables

**Table 1:** Theoretical ATP generation rate calculated from glycolytic fluxes or TCA cycle fluxes, respectively. Data are presented as median, and the interquartile range is indicated within parenthesis. Fluxes were multiplied with the corresponding factor (2.5 ATP per NADH, 1.5 ATP per FADH_2_, 1 ATP per GTP [40]): hexose uptake: −1 (−1 ATP); ΔQ2: −1 (−1 ATP); Q11: 4.5 (2 ATP + 1 NADH); lactate production: −2.5 (−1 NADH); F2: 2.5 (1 NADH); F4: 2.5 (1 NADH); F3: 7.5 (1 GTP, 1 FADH_2_, 2 NADH); F5: 1 (1 ATP); F6: −1 (−1 ATP). It should be noted that the net utilization of ATP (negative values) via glycolysis can be compensated for by ATP production in the TCA cycle.

		Theoretical ATP Generation Rate (nmol/2 h/10^6^ Cells)	
		PBMCs	Granulocytes
Glycolysis	both sexes (*n* = 12)	13.8 (4.3; 23.1)	49.3 (15.1; 64.8)
(hexose uptake, ∆Q2, Q11, lactate production)	female (*n* = 6)	1.9 (−19.9; 18.8)	54.3 (23.7; 65.6)
	male (*n* = 6)	14.4 (13.6; 21.6)	43.6 (15.5; 55.8)
TCA cycle	both sexes (*n* = 12)	30.7 (28.5; 34.1)	14.2 (12.2; 17.3)
(F2, F4, F3)	female (*n* = 6)	30.7 (29.0; 31.9)	14.2 (12.7; 16.5)
	male (*n* = 6)	47.9 (44.5; 51.1)	14.9 (12.1; 17.8)
Whole metabolism	both sexes (*n* = 12)	45.4 (36.4; 53.0)	62.5 (29.9; 78.6)
(hexose uptake, ∆Q2, Q11, lactate production, F2, F3, F4, F5, F6)	female (*n* = 6)	34.0 (7.3; 52.0)	68.8 (37.9; 79.1)
	male (*n* = 6)	47.9 (44.5; 51.1)	58.5 (33.0; 71.3)

**Table 2:** Metabolic fluxes that contributed significantly to the first three principal components (PC) of the PCA. Contribution of each PC to the total variance is indicated within parenthesis. The first three PCs amount to ~81% of the variance. The signs indicate the direction of the PC relative to the mean.

Metabolic Flux/Effector Function		PC1 (37.4%)	PC2 (23.7%)	PC3 (20.0%)
Effector function	O_2_^•−^ production	0.67		−0.61
Mitochondrial respiration	*J*O_2_ of ROUTINE respiration			0.63
Glycolysis	hexose (glucose/fructose) uptake	0.59	0.66	−0.36
	∆Q2 (F6P → GAP+ DHAP)	0.89	0.26	
	Q11 (GAP → Pyr)	0.91		
	lactate production	0.92		
Oxidative PPP	Z3	0.87		
Non-oxidative PPP	∆TAL (F6P + E4P → S7P + GAP)		−0.88	0.39
	∆TKT1 (S7P + GAP → X5P + R5P)		−0.99	
	input S7P		0.64	0.55
	Q4 (R5P loss)		0.97	
TCA cycle	F2 (Pyr → AcCoA + CO_2_)	−0.56		
	F3 (Akg → Oac + CO_2_)	−0.78		0.49
	F4 (Oac + AcCoA → Akg + CO_2_)	−0.80		
	F6 (Pyr + CO_2_ → Oac)			0.75
	ΔAsp (Asp_Input_ − Asp_Loss_)			−0.78
	glutamine input	−0.68		0.62

**Table 3:** Sex-specific correlations (Spearman/Pearson) between metabolic fluxes of granulocytes [nmol/10^6^ cells/2 h] and aldosterone plasma levels [pg/mL plasma]. Aldosterone plasma levels were determined by ELISA and metabolic fluxes by ^13^C-MFA, where posterior mean was used for evaluation. Differences with *p* < 0.05 were considered as significant and are highlighted in bold and marked by *.

Plasma Level [pg/mL] vs. Metabolic Flux [nmol/2 h/10^6^ Granulocytes]	Sex	Spearman (*n* = 6)		Pearson (*n* = 6)	
		r	*p*	r	*p*
Aldosterone vs. hexose uptake	female	−0.886	0.033 *	−0.949	0.004 *
	male	0.886	0.033 *	0.609	0.200
Aldosterone vs. ΔTAL	female	1.000	0.003 *	0.916	0.010 *
	male	−0.657	0.175	non-normal distribution	
Aldosterone vs. ΔTKT1	female	0.904	0.013 *	0.904	0.013 *
	male	−0.726	0.102	−0.726	0.102
Aldosterone vs. input S7P	female	−0.543	0.297	−0.664	0.150
	male	1.000	0.003 *	non-normal distribution	
Aldosterone vs. R5P loss (Q4)	female	−0.943	0.017 *	−0.917	0.010 *
	male	0.714	0.136	0.672	0.144

## Data Availability

All the row data of this article will be made available by the authors upon request.

## Supplementary Materials

The following supporting information can be downloaded at: https://www.mdpi.com/article/10.3390/biom14010098/s1, Figure S1: Flux relations in PBMCs and granulocytes; Excel file: Supplementary material.xlsx.
