# Supplementary material for: Ex Vivo 13C-Metabolic Flux Analysis of Porcine Circulating Immune Cells Reveals Cell Type-Specific Metabolic Patterns and Sex Differences in the Pentose Phosphate Pathway
Source: Biomolecules. 2024 Jan 12;14(1):98. doi: 10.3390/biom14010098 (PMC10813356; doi:10.3390/biom14010098)
Supplement: Supplementary file 1 [file biomolecules-14-00098-s001.zip › Figure S1.pdf]

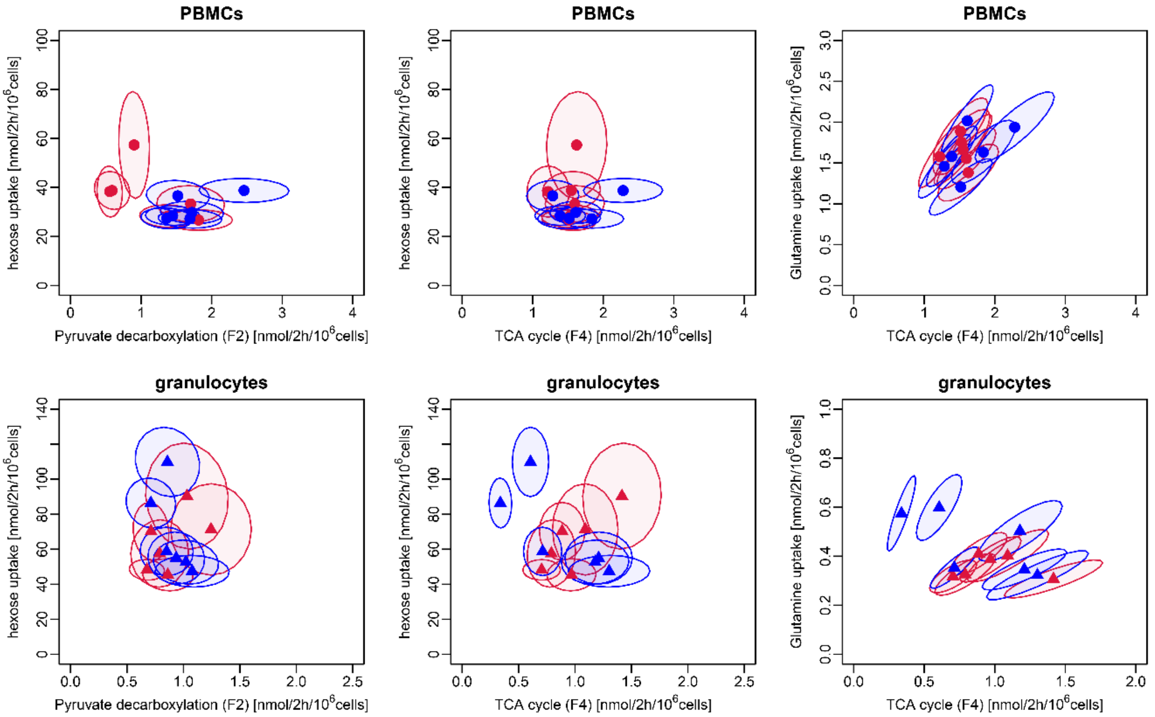

Figure S1. Flux relations in PBMCs and granulocytes: Left/middle: Relations between hexose (glucose/fructose) uptake rate and F2 (Pyruvate → AcCoA + CO_2), and hexose (glucose/fructose) uptake and F4 (Oac + AcCoA→ Cit→ Akg + CO_2), respectively. Right: Correlation between glutamine uptake into the TCA cycle and TCA cycle flux F4 (Oac + AcCoA→ Cit→ Akg + CO_2). Bayesian ^{13}C-MFA results are displayed as an ellipse with 68% confidence interval (± one standard deviation of the mean) of posterior distributions with posterior mean indicated by symbols (PBMCs: circles, granulocytes: triangles). Data from female pigs are highlighted in red and males in blue.
